# Honey Bee Viruses in Wild Bees: Viral Prevalence, Loads, and Experimental Inoculation

**DOI:** 10.1371/journal.pone.0166190

**Published:** 2016-11-10

**Authors:** Adam G. Dolezal, Stephen D. Hendrix, Nicole A. Scavo, Jimena Carrillo-Tripp, Mary A. Harris, M. Joseph Wheelock, Matthew E. O’Neal, Amy L. Toth

**Affiliations:** 1 Department of Ecology, Evolution, and Organismal Biology, Iowa State University, Ames, Iowa, United States of America; 2 Department of Biology, University of Iowa, Iowa City, Iowa, United States of America; 3 Department of Plant Pathology and Microbiology, Iowa State University, Ames, Iowa, United States of America; 4 Department of Natural Resource Ecology and Management, Iowa State University, Ames, Iowa, United States of America; 5 Department of Entomology, Iowa State University, Ames, Iowa, United States of America; University of North Carolina at Greensboro, UNITED STATES

## Abstract

Evidence of inter-species pathogen transmission from managed to wild bees has sparked concern that emerging diseases could be causing or exacerbating wild bee declines. While some pathogens, like RNA viruses, have been found in pollen and wild bees, the threat these viruses pose to wild bees is largely unknown. Here, we tested 169 bees, representing 4 families and 8 genera, for five common honey bee (*Apis mellifera*) viruses, finding that more than 80% of wild bees harbored at least one virus. We also quantified virus titers in these bees, providing, for the first time, an assessment of viral load in a broad spectrum of wild bees. Although virus detection was very common, virus levels in the wild bees were minimal—similar to or lower than foraging honey bees and substantially lower than honey bees collected from hives. Furthermore, when we experimentally inoculated adults of two different bee species (*Megachile rotundata* and *Colletes inaequalis*) with a mixture of common viruses that is lethal to honey bees, we saw no effect on short term survival. Overall, we found that honey bee RNA viruses can be commonly detected at low levels in many wild bee species, but we found no evidence that these pathogens cause elevated short-term mortality effects. However, more work on these viruses is greatly needed to assess effects on additional bee species and life stages.

## Introduction

Pollinating bees provide a key ecosystem service in both natural and agricultural environments [1, 2]. In much of the world, honey bees are the major managed pollinator [3], but wild bees can also improve yields of various crops and are a key part of ecosystems [1, 4]. Recently, however, there have been declines in both managed and unmanaged bee populations [5, 6], likely as a result of multiple interacting factors including habitat losses, pesticide exposure, and pathogen/parasite pressure [7]. Honey bees, arguably the best studied pollinator, are host to numerous parasites and pathogens that can have severe impacts on their health [8], with virus infections of particular importance [9–11]. Honey bee viral infections have the potential to cause, or contribute to, colony mortality [9, 12], are correlated with colony collapse disorder (CCD) [13], and can be spread through numerous routes [14].

There has been recent concern that common honey bee viruses can spread to other bee species, contributing to their declines [11, 15–19]. While there is evidence of some pathogen and parasite transmission from managed to wild bee populations [15, 18, 20], the potential effects of virus infections are largely unknown. Viruses, particularly fast-evolving RNA viruses with many variants, also have a high propensity for shifting hosts, allowing them to emerge quickly in new host species [10, 11, 16, 18, 19]. Therefore, exposure of wild bee species to emerging pathogens from the managed honey bees [11], and the potential threat such viruses pose to wild bees, needs investigation.

Of the 24 RNA viruses currently identified in honey bees [14, 17], some may have a wider host range than just honey bees; notably, deformed wing virus, (DWV) may infect many bee species, as well as a diverse group of other insects, including flies, beetles, ants [16, 21–24]. Singh et al. [25] detected black queen cell virus (BQCV), DWV, Israeli acute paralysis virus (IAPV), Kashmir bee virus (KBV), and sacbrood virus (SBV) in multiple bee and wasp species. Most notably, many honey bee viruses have been routinely detected in bumble bees [18, 25–27] where they cause detrimental effects on development, lifespan, and colony health [28–30]. Spread of these viruses to bumble bees likely occurs from direct or indirect contact with infected honey bees. For example, when honey bees are kept nearby, identical strains of deformed wing virus (DWV), arguably the most widespread honey bee virus, are more prevalent in bumble bee populations, suggesting intergeneric transmission [28]. IAPV, a honey bee virus linked to CCD symptoms [13], also may be transmitted from honey bees to bumble bees, and vice versa, through shared floral resources [25, 31] and DWV transmission was reported when bumble bees rob honey bee nests [21, 26].

While these studies suggest that DWV and IAPV can replicate in some non-honey bee species [18, 22, 28], there are still important gaps in understanding of how honey bee viruses may infect other bees [16, 19]. First, the majority of the previous work used bumble bees [16, 18, 19, 26, 28, 32], which are in the same family as honey bees (Apidae) and are also eusocial. However, there are potentially hundreds or thousands of other bee species from different families coming into contact with honey bee viruses and little is known about their exposure or response to these pathogens. Second, the few previous studies of non-Apidae have only tested for the presence of viruses–they do not put the virus levels detected in a context that allows for assessment of health risk. Finally, it has not been thoroughly explored whether presence of honey bee viruses in other bee species, especially non-Apidae, results in deleterious effects, such as increased mortality, or if the viruses are even able to replicate in other hosts [16, 19].

To begin to address these gaps, we present the first biologically relevant and quantitative analysis of the viral load and effects of common honey bee viruses in multiple species of wild bees spanning both social and solitary species. We collected wild bees from 15 species representing 4 families at five agricultural and prairie sites in Iowa, USA, an area of high agricultural intensity and risk concern for pollinator health [32]. We then measured the incidence of five common honey bee viruses in wild bees. We also quantified virus titers in these bees and compared these levels to honey bee foragers caught in the same locations and to honey bees from a nearby, managed apiary. To determine the potential source of these viruses, we also explored whether honey bee presence at our collection sites was related to detection and titer of viruses in wild bees. To evaluate the effects of honey bee virus occurrence on non-honey bee, non-Apidae species, we examined mortality and virus level changes in two solitary bee species (*Colletes inaequalis* and *Megachile rotundata*) after treatment with viral inoculum known to cause substantial mortality in honey bees [33]. Together, these data provide critically needed information about honey bee virus incidence, load, and mortality induction in wild bees.

## Materials and Methods

### Bee Collection and Identification for Survey of Virus Titers

Bees were collected from five sites in Iowa, USA between May 14-August 6, 2013: three remnant tallgrass prairies (Cayler Prairie Preserve, Anderson Prairie State Preserve, Doolittle Prairie State Preserve), a restored tallgrass prairie (Neal Smith Wildlife Refuge), and a commercial soybean field in Story County (S1 Table). For Cayler, Anderson, and Doolittle, permission to collect was granted by the Iowa State Preserves Advisory Board; for Neal Smith, permission was granted by the National Fish and Wildlife Service; collections at commercial soybean fields were performed with permission of private landowners. For each site, permission was arranged through Iowa Lakeside Lab, was not required, or was performed with the approval of private landowners. No threatened or endangered species were used in this study. At the first four sites, bees were collected from abundantly blooming forbs by netting in a 30–60 minute period [34]. In the agricultural site, bees were collected in modified pan traps (i.e., bee bowls containing soapy water) [35]. The use of multiple field sites of different land use types across the season allowed for capture of a wide variety of bee species. Bees were collected live into tubes kept on wet ice in the field for <3 hours. In both bee bowls and nets, it is possible that bees contacted each other, resulting in some surface-level cross contamination, though internal cross-contamination is very unlikely.

Once returned to the lab, collected bees were placed in a -20°C freezer until identification, within 24 hours. For netted samples, detailed identification was performed to genus using Michener [36, 37] and to species, if possible, using Mitchell [38, 39] and monographic keys. Bees were kept on ice as much as possible during identification. Bees collected in bee bowl traps were transported back to the laboratory, processed [40] and identified using the Discover Life key [41]. After visual identification, bees were punctured and immersed in Trizol reagent (Thermofisher) and then stored at -20°C onsite at Iowa Lakeside lab; within 3 days, they were transported to Iowa State University and kept at -80°C until processing for virus quantification using RT-qPCR. To address the possibility of differential viral RNA degradation between the two collection methods, we compared virus titers in the best-represented bee family (Halictidae) across collection types. We found no significant differences in virus titers related to collection method, indicating that it is unlikely that collection method biased our results (S1 Fig).

To allow for a reference point for virus levels, samples were also taken from managed honey bee hives at the Iowa State University research apiary. Because these viruses are found commonly in managed honey bee hives, this sample allows us to place wild bee virus titers into context with those of honey bees without apparent pathogenic effects, i.e. levels in apparently healthy virus “carriers”. In July 2013, during the same time period during which we made wild bee collections, live adult workers were sampled from the brood nest (center) of 25 honey bee colonies. The majority (n = 15) of these colonies either survived the winter or were made from splits of these surviving colonies; the others (n = 10) were made from commercially-purchased packages. Splits or packages were established 3 months prior and were regularly inspected for overt pathogen symptoms and general health throughout the season [42]. All colonies appeared healthy and continued to grow and survive through the rest of 2013. To ensure the sample represented the hives as a whole, approximately 30mL of bees were collected from the brood nest and honey frames of these hives on wet ice (keeping the bees alive), transported to the laboratory, then stored at -80°C until processing. Hive representative samples were then created by pulverizing the 30mL collection of bees in liquid nitrogen. From this homogenate, 0.3 grams (the approximate mass of three adult bees) was then analyzed for virus levels using RTq-PCR. This approach, measuring a homogenate made of many bees, allows for a higher chance of detecting a virus present in the hive and a more representative characterization of the virus presence at the hive level.

### Inoculum Preparation

The viral inoculum used here was produced by the methods of Carrillo-Tripp et al. [33]. Note that the percent makeup described here is slightly different than in Carrillo-Tripp et al.; variations occur in aliquots of the virus mixture due to storage time and aliquot. The viral stocks used here were stored for less time than those described in Carrillo-Tripp et al, and were stored as different stock aliquots. The makeup of the viral inoculum at the time of this experiment was quantified as consisting of SBV (89.93%), IAPV (9.68%), DWV (1.2%), and BQCV (0.17%). This inoculum consists of a mix of viral species naturally occurring in honey bee colonies in the field. It is highly lethal to honey bees and, while SBV is the main component, results in significant elevation in IAPV titer after just 48 hours, showing that, when honey bees are fed this inoculum, IAPV replicates very quickly (within 48 hours), and is thus the likely the main virus causing high observed mortality [33].

### Determination of Viral Inoculum Lethality in Caged Honey Bees

To determine the dosage of viral inoculum necessary to cause mortality in honey bees, cages of 35 newly emerged honey bees were orally infected with the inoculum. Each cage of bees received an open feeder containing 0.6mL 30% sucrose solution (control) or a serial dilution of the viral inoculum stock, with a total of 5 cages receiving each dose as follows: undiluted stock, 1:10, 1:100; 1:1000; 1:10,000, 1:100,000. Estimated genome equivalents of each virus in 1 μl of undiluted stock are as follows: BQCV: 1.02E+06; DWV: 7.06E+06; IAPV: 5.68E+07; SBV: 5.21E+08. Cages were randomly distributed throughout the chamber during the experiment. Bees had *ad libitum* access to the feeders for 12 hours, during which all of the solution was consumed in all cages. Afterwards, each cage received *ad libitum* access to untreated sucrose solution. Mortality was measured in each cage daily for 5 days. Based on these results (S2 Fig), the 1:1000 dilution of virus stock was chosen for solitary bee infections, as it was the lowest virus dose that caused approximately 100% mortality in honey bee cages.

### Acquisition of Live Solitary Bees for Inoculation Experiments

To evaluate the effects of an extreme dose of honey bee virus on solitary bees, two different non-Apidae bee species were chosen for experimental inoculation: lab reared *Megachile rotundata* and locally collected *Colletes inaequalis*. For experiments, *M*. *rotundata* were acquired as newly emerged adults from the USDA North Central Regional Plant Introduction Station, Ames, IA, USA, where they are used for pollination services. The difficulty in testing non-managed native bees lies in acquiring enough bees at the same life stage with confidence that a single species is being collected, as many bees are difficult to identify. To ensure only one species of bees of approximately the same age were used, dense nesting sites of *Colletes inaequalis* (Colletidae) were identified in preceding years, and observed as the first bees were emerging in the spring in these areas. Careful observation of their nesting sites in the early spring allowed for collections directly after emergence, ensuring bees were early in their adult life stage and reducing the chances of collecting other species of bees. Individual *C*. *inaequalis* bees were identified on sight in or near Brookside Park, Ames, IA, USA (42.031203, -93.629722) using sweep nets to collect adults as they left their nests or landed on flowers. Nesting areas were observed regularly before bees were present, and bees were collected upon first observation, implying that all collected bees were early in their adult life cycle. In the laboratory, visual inspection of the bees verified that bees matched voucher specimens, which was identified and verified with the Discover Life Key [41]. Because *C*. *inaequalis* individuals were collected directly from the field, it was not possible to coordinate all trials to begin together on a single day. Instead, bees were collected as they were available from the field over a 17 day period.

### Experimental Inoculation of Live *M*. *rotundata* and *C*. *inaequalis* Bees

On the day of acquisition, each bee was kept alone inside a sterile, 17x100mm polystyrene culture tube with an aerobic cap inside of an incubator kept at approximately 30°C. Each bee received a cotton wick soaked in 0.5 mL of 1:1000 dilution of viral inoculum in 30% sucrose solution or 0.5 mL of heat inactivated viral inoculum in 30% sucrose solution. Heat-inactivated virus was produced by heating aliquots of viral stocks to 95°C for 30 minutes, providing a negative control [33]. While not measured quantitatively, bees were regularly observed feeding on the sugar solution in the cotton wicks. At the end of the experiment, the wicks were noticeably damaged from feeding/chewing. The bees received no other food or water source during the experiment. After 24 hours, 6 bees were collected for analysis of 1 day-post-infection virus levels. Wicks soaked in untreated 30% sucrose solution were then used to replace the treatment wicks. Mortality was observed daily for a total of 5 days. Dead bees were placed into 1.5 mL centrifuge tubes and stored at -80°C. After five days, all remaining live bees were collected by placing the culture tubes directly into a -80 °C for 24 hours, then transferred to 1.5 mL centrifuge tubes for long term storage at -80 °C. Virus titers at 1 day-post-infection and 5 days-post-infection were then be measured via RTq-PCR.

### Virus Quantification

For both the field-collected bees and the bees used for experimental infection studies, total RNA was extracted each bee sample using Trizol (Thermofisher), treated with DNAse I, and diluted to 100 ng/μl. In the case of very large bees (e.g., bumble bees), the whole body was homogenized, then a subset of the tissue was used for extraction. Genome copy number of five viruses commonly detected in other studies and in our routine screenings of the Iowa State University research apiary was determined by absolute quantification approach by extrapolation to a standard curve of *in vitro* synthesized viral RNA, as described in Carrillo-Tripp et al. [33]. These five viruses were black queen cell virus (BQCV), Israeli acute paralysis virus (IAPV), deformed wing virus (DWV), sacbrood virus (SBV), and Lake Sinai virus (LSV). All of these were detected with previously published primers [33], with the except of LSV, which were as follows: forward: TGCGGACCTCATTTCTTCATGT and reverse: ATTCCGTCGATAGACCAGC. For each target, a dynamic range, which defines quantitation limits (i.e., a limit of detection) based on multiple tests of serial dilutions of a standard curve [14], was produced and calculated using Bio-Rad CFX software. Data out of the dynamic range for each target were considered below detection limits and treated as “undetected”. The minimum limit of detection for SBV, LSV, and IAPV were 4.92E+02 viral genome equivalents and for DWV and SBV 4.92E+03 viral genome equivalents, as determined with a Universal Standard Reference by Carrillo-Tripp et al. [33]. Melting curves of qPCR products were as expected and matched the viral RNA positive controls.

### Statistical Analysis

Virus incidence data from field-collected bees were analyzed on the family level. After assigning each bee to its corresponding family level classification (Andrenidae, Apidae (excluding honey bees), Halictidae, and Megachilidae), we calculated the proportion of individuals with detectable levels of BQCV, DWV, IAPV, LSV, and SBV from each family and compared this to the incidence of detection of these viruses in field-collected honey bees and apiary-collected honey bee samples by χ^2^ analysis with α = 0.05 using JMP Pro 11. For comparisons of virus titers between wild bees and honey bees, only bees where viral titers exceeded limits of detection were included (i.e., ‘undetectable’ levels, which would be considered a titer of zero, were not included). For the purposes of statistical testing, data were pooled by family, and only bee families with a usable sample size of bees were included, resulting in the exclusion of Colletidae. Titer comparisons were made using a mixed model ANOVA by the ‘lme’ function from the R package ‘nlme’, followed by a Tukey posthoc test from the R package ‘multcomp’. Virus titers were log transformed to normalize the distribution of the data.

The effects of different dilutions of viral inoculum on caged honey bees were evaluated by a one-way ANOVA followed by a Tukey HSD posthoc test using JMP Pro 11. Mortality of solitary bees treated with virus inoculum was compared to heat-inactivated virus treated bees by χ^2^ analysis (α = 0.05) using JMP Pro 11. Virus titers of these bees was compared using the lm function in R followed by ANOVA.

## Results and Discussion

### Wild Bees Frequently Harbor Honey Bee Viruses

In total, 284 bee specimens were collected and identified to species from four prairie sites and one agricultural site in Iowa, USA, representing 26 species, 11 bee genera from 5 bee families: Andrenidae (n = 54), Apidae (n = 80; 21 of which were *A*. *mellifera*), Colletidae (n = 3), Halictidae (n = 124), and Megachilidae (n = 23). The most common genera collected were *Halictus* (n = 56), *Andrena* (n = 53), *Bombus* (n = 36), *Agapostemon* (n = 29), *Apis* (n = 24), and *Megachile* (n = 23). We measured virus titers in genera with at least 5 individuals collected and analyzed a maximum 10 individuals of a given genus per site, resulting in a total of 148 wild bees and 21 wild-caught honey bees representing 17 species, 8 genera, and 4 families (S2 Table).

Of the 148 non-honey bees collected, 80.4% had detectable levels of at least one honey bee virus confirming previous reports that wild bees are exposed to and commonly found carrying honey bee viruses [16, 19, 21, 22, 24, 25]. Overall, DWV and SBV were the most commonly detected viruses in non-honey bees, with 53% and 45% of specimens showing the presence of each of these viruses, respectively. IAPV was detected in over 20% of non-honey bees, Lake Sinai virus (LSV) in over 12%, and BQCV with only around 3% showing detectable levels. In non-honey bees, BQCV and LSV were rare, even though these two viruses were very common in both foraging honey bees and apiary-collected hive bees. On the other hand, DWV was similar common across species, occurring at the same frequency in wild bees, field-collected honey bees, and hive bees. It is perhaps not surprising that DWV would be detected frequently in wild bees, as it was found in previous surveys of wild bees and other pollinators, and there is already evidence that it has a more general host range [16, 26].

**Fig 1:**
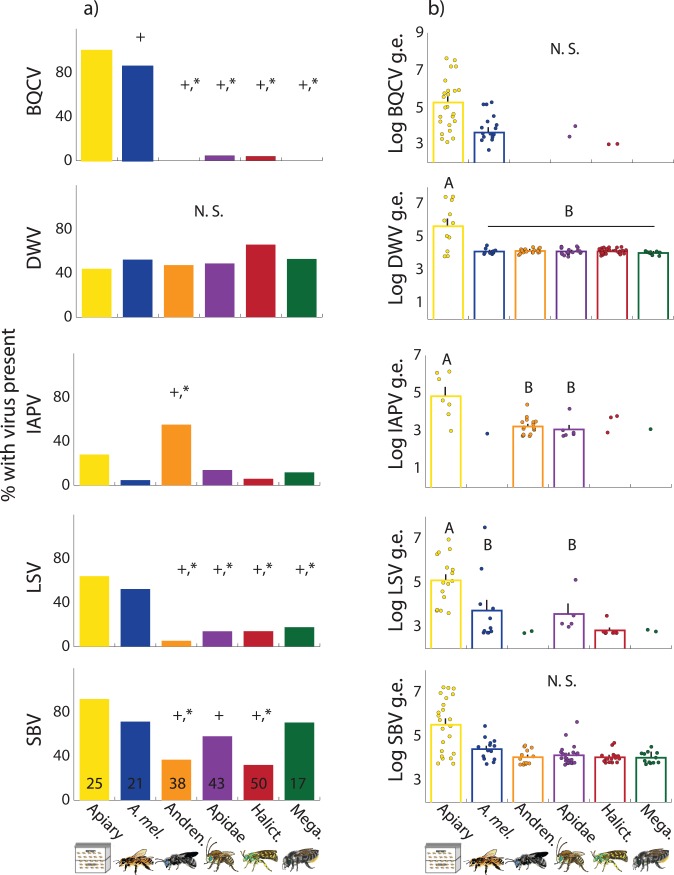
a) Percentage of each bee group (family or honey bee group) detected with BQCV, DWV, IAPV, LSV, SBV. Sample sizes indicated, * denotes significantly different from field-collected honey bees, ⁺ denotes significantly different from apiary-collected honey bee samples (χ^2^, p<0.05). b) Log genome equivalents (g.e; an estimate viral genome copy number in 200 ng of total RNA) of BQCV, DWV, IAPV, LSV, and SBV in bees with detectable levels of each virus. Plot shows each individual data point for each bee family, but means +s.e. are only shown for families with enough positive detections for statistical comparisons. Letters denote significant differences (ANOVA, Tukey HSD, p<0.05).

Occurrences of some viruses varied greatly between bee families and honey bees versus non-honey bees (Fig 1A). SBV was most commonly detected in apiary-collected honey bees, though presence of SBV in wild bee samples was also quite frequent, particularly in the Megachilidae, where presence was similar to that in honey bees. IAPV was significantly more likely to be present in Andrenidae compared to either honey bee type, but it is not clear why they have such a high IAPV presence compared to other wild bees and honey bees, either foraging or in the apiary. These results do suggest that, even when a virus is not commonly detected in honey bees, it may be common, at least at a local scale, in wild bees (Fig 1A, S3 Table).

### Viral Load Is Low in Wild Bees

These common detections of honey bee viruses in other potential bee hosts support the findings of other studies showing that viruses can have inter-species transmission within the pollinator community [21, 23–25]. However, previous research outside of the family Apidae has mainly screened for the presence of viruses in other insects; they have not quantified virus levels and analyzed them in the context of those found in honey bees [16]. Without this comparison, it is difficult to conceptualize the intensity of infection or exposure [43]. Therefore, we compared virus levels of all bees with levels above the limit of detection (i.e., those with undetectably low virus levels were not included) to levels found in honey bees with detectable virus levels. This approach puts virus detection in a more relevant biological context, and provides important new information on whether honey bee viruses are present in wild bees at levels likely to have consequences for their health.

Across all bee families, levels of honey bee viruses were extremely low in wild bees relative to foraging or apiary-collected honey bees (Fig 1B). In some cases, detection of a virus was too rare in wild bees to allow for statistical comparisons (i.e., where only 1–3 individuals in a family had detectable levels), but we graphically present all these data to illustrate that virus levels were generally low in wild bees. For example, BQCV was extremely rare in wild bees, and levels were not different between wild-collected honey bee foragers and apiary samples. While DWV was very common across all groups, we found that the virus levels of wild bees detected with DWV were significantly lower than those found in apparently healthy apiary colonies. No difference in DWV levels were detected between wild bees and honey bee foragers caught in the field, but all field-collected honey bees showed DWV levels, on average, two orders of magnitude lower than those collected in the hives. A similar pattern was observed in IAPV and LSV. Overall, these data show that, while wild bees are detected with multiple honey bee viruses at the same frequency and even the same levels as honey bee foragers collected in the field, the levels are substantially lower than those found inside healthy hives (Fig 1B). Furthermore, for IAPV, the levels were three orders of magnitude lower than levels found in honey bees experiencing IAPV-induced mortality [33]. Therefore, while the high frequency of virus detection could appear alarming, the actual virus load for every wild bee specimen we examined was substantially lower than the levels found in healthy honey bee colonies. The comparison of wild bees with hive-collected honey bees allows a comparison baseline of virus titers that have no apparent effect at the honey bee hive level. These data suggest that, while many wild bees are coming in contact with these viruses, virus titers were always detected at extremely low levels (Fig 1B; S4 Table). However, we note that there is substantial variability in the virus titer necessary to cause symptomatic effects in honey bees [44], and we know next to nothing about this in other potential hosts. Therefore, it is not possible to infer that low virus titers equates with low virulence. This is particularly notable in the context of potential strain variation in viral species. Previous work has shown spread of specific viral strains between honey bees and bumble bees [18, 28], and it is well-known that different strain variants exist for multiple honey bee viruses [45, 46]. Here, we used a detection method optimized for virus strains commonly found in honey bees; it is possible, however, that other strains exist in wild bee species that would be less likely to be detected, and that could have different virulence. While outside the scope of this study, future work sequencing variants found in wild bee species would be useful to better understand these dynamics.

### Extremely High Doses of Virus Inoculum Cause Severe Mortality in Honey Bees but Not Wild Bees

Although our data clearly show virus levels in wild bees were substantially lower than those found in healthy honey bee hives (Fig 1B), these data alone are inconclusive for assessing health risk to wild bees. Bees collected for virus measurements were apparently healthy (flying and foraging on flowers) but may be a sub-set of a community that contains other more highly-infected bees too ill to leave their nests, or could have died very quickly after exposure [16]. Also, the virus titers found in free-flying bees could be low for honey bees, but still cause health effects in other species. Whereas detection of a virus simply shows that a bee has come in contact with virus particles, for example, by consuming contaminated pollen [16], infection experiments test whether viruses can replicate, infect, and have health effects on hosts. Previous studies, particularly in other bees closely related to *A*. *mellifera*, have used detection of negative-strand RNA (a replication intermediate of positive strand RNA viruses) to infer viral replication [22, 28, 47], but infection experiments on non-Apidae are sorely lacking [16].

**Fig 2:**
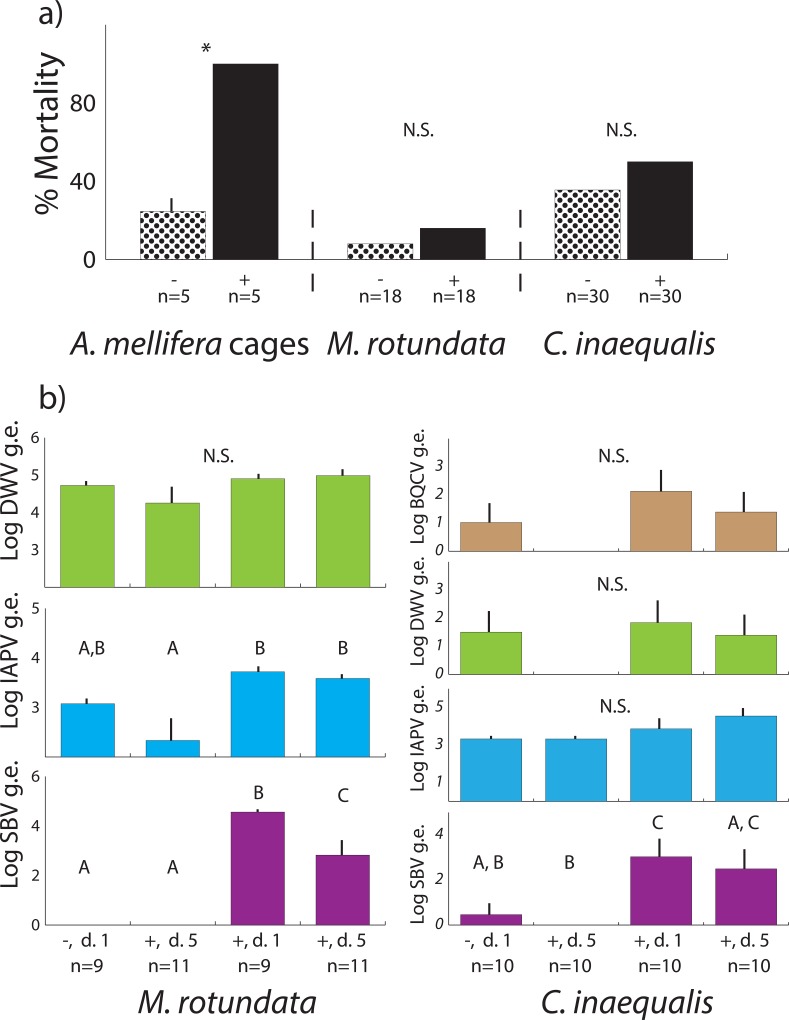
a) Mean +s.e. percent mortality in cages of honey bees, *M*. *rotundata* and *C*. *inaequalis*, treated with control (-) or virus (+) after 5 days. * denotes significant differences, N.S. = not significant. For honey bee cage comparison: ANOVA, Tukey HSD, p<0.05; *M*. *rotundata* and *C*. *inaequalis* (χ^2^, p<0.05). Sample sizes indicated. b) Log + s.e. of detectable virus genome equivalents (g.e.) of control (-) or virus treated (+) *M*. *rotundata* and *C*. *inaequalis* collected at 1 and 5 days post exposure (d). Sample sizes indicated, letters denote significant differences. N.S. = not significant.

To fill this gap in our knowledge, we used an experimental approach to expose two representative non-Apidae solitary bees to a dose of oral viral inoculum known to cause substantial mortality in honey bees. This inoculum consists of a mixture of viruses, mostly of SBV and IAPV with low levels of BQCV and DWV. It was produced by infecting honey bee pupae with virus particles extracted from infected hives, allowing the virus levels to increase until the pupa died, and then purifying and concentrating the virus particles, resulting in extremely concentrated and potent viral mixture [33]. Within three days of oral exposure to the inoculum, newly emerged honey bees show very high levels of mortality (Fig 2A, S1 Fig, [33]). While SBV is a major part of the mixture, IAPV replicates very quickly to contribute to honey bee mortality [33]: bees that die after exposure have extremely elevated levels of IAPV, but no other viruses. This powerful virus inoculum provides the opportunity to test the response of wild bees to an extremely high level of honey bee virus exposure–much higher than they could likely be exposed to in the field.

We fed the virus inoculum to commercially-reared solitary bees, *Megachile rotundata* (Megachilidae) and field-collected *Colletes inaequalis* (Colletidae), in a controlled laboratory setting. These bee species were chosen for availability and because they represent two non-Apidae bee families. Even in honey bees, viral titers and responses are highly variable by age [48], and therefore it is imperative to test pathogen response only in similarly-aged individuals. Using these bee species allowed us to collect and test bees at a similar life stage, specifically, shortly after adult emergence.

Each treated *M*. *rotundata* and *C*. *inaequalis* received a dose of a) viral inoculum that caused approximately 100% mortality in caged honey bees or b) a control treatment consisting of the same viral mixture that had been inactivated by heat [33]. Bees were provided this food *ad libitum*, with no other food or water source available. Unlike honey bees, when these solitary bees were exposed to the inoculum, no differences in mortality were observed compared to controls over a 5 day period (Fig 2A; S5 Table). Our experimental design allowed us to verify that the bees were exposed to the viral inoculum, as SBV levels were substantially higher in treated bees compared to controls (Fig 2B). This could be due to a mixture of oral exposure (i.e., inoculum that entered the bees) and topical exposure (i.e., inoculum present on the cuticle of the bee), but it clearly shows that these bees came in contact with high levels of virus. However, because levels did not increase over time, and very few bees died, our data show no evidence that these viruses cause extreme adult mortality over a short time span, as they do in honey bees [33].

In *M*. *rotundata*, SBV levels even decreased from 1 day post exposure to 5 days post exposure, suggesting degradation of the virus over time. Furthermore, the virus levels observed in both *M*. *rotundata* and *C*. *inaequalis* were several orders of magnitude lower than those found in inoculated honey bees [33] and were still lower or not different than the levels found in foraging honey bees or samples from healthy apiary hives (S3 Fig, S6 Table). Therefore, our data provide no evidence that these viruses can replicate or cause mortality in either of these solitary bees. While it is possible that the solitary bees tested here consumed less of the inoculum than caged honey bees do, and therefore received a lower dose, the viral inoculum was the only food provided to the bees and it was provided *ad libitum*. Even at concentrations 100-fold lower than those used in this experiment, the virus mixture causes over 60% mortality in honey bees (S2 Fig). In addition, honey bees healthy enough to spread disease by foraging and visiting shared floral resources would likely not have viral contamination levels this high, though somewhat higher levels might be possible if other bees kleptoparasitize honey bee hives that have collapsed due to viral infection. Despite the use of this unnaturally high dose, no elevated mortality was observed in either solitary bee species. It is worth noting, however, that the virus inoculum was derived from honey bees, and likely contained viral strain variants that are more adapted to infection of honey bees. It is possible that other strain variants of these viruses exist within wild bee populations that would show higher virulence in other species. Future research is necessary to study the variation in virus populations and if different variants exist and exhibit difference virulence in different species.

Although the findings here may appear to reduce concern about the detrimental effects of viruses on wild bees, it is premature to dismiss the issue, as our data do not yet rule out the possibility that some honey bee viruses can infect and harm some species of wild bees [16]. Many more species of bees and pathogens need to be tested, and longer-term studies are needed to test potential effects of pathogens on lifespan, behavior, or reproduction. [26]. There is also the potential that other, non-viral, bee pathogens could affect wild bee populations [21, 49–51]; if wild bees are coming into contact with honey bee viruses so regularly, it is likely that other pathogens are spread similarly. Furthermore, RNA viruses have a high propensity for host shifting [52], and continued high-frequency contact with quickly-evolving honey bee viruses could result in increased risk that these viruses could evolve new levels of virulence or transmission, especially towards more closely-related bees, e.g. Apidae [16, 19]. Furthermore, our findings from both wild bee populations and laboratory-inoculated bees show that these pathogens are likely to be persistent in the environment. Even if a given bee species is not susceptible to infection from a virus, it could still spread infections between honey bee colonies through shared floral resources [15, 25, 53] and providing ample opportunities for future exposure and host shifting in wild bees. Furthermore, while previous research has focused on transmission of pathogens from managed bees to wild bees, little work has been done to study if there is bi-directionality in pathogen transmission. It is possible that some pathogens are transmitted, not just from honey bees to wild bees, but vice versa.

Overall, it is imperative that more work be done to continue monitoring pathogen transmission from and/or into wild bee populations. Most importantly, it is critical that more experiments be done to experimentally infect and test the effects of honey bee viruses on other bee species, with more rigorous investigations of their effects on different life stages and in conjunction with other stressors. While our data show no substantial mortality effects over a short time, it is very possible that longer term effects on lifespan may occur or that sublethal viral infection could affect reproduction, behavior, or overwintering success.

## Supporting Information

S1 Fig Mean +/- s.e. Log genome equivalents (g.e.), with each data points shown, of DWV, IAPV, LSV, and SBV comparing virus titers above detection of the bee family that was most abundant with each collection method (bee bowls in crops vs. netting in prairies; Halictidae for DWV, LSV, and SBV; Apidae for IAPV). BQCV was too rarely detected for comparisons. Virus titers were not significantly different between any groups (t-test, p<0.05). This suggests that, even though samples had slightly different treatments due to collection methods, there was no significant effect on virus titer because of this.
(TIF)

S2 Fig Mean +/- s.e. percent mortality in cages of honey bees treated with different dilutions of viral inoculum. N = 5 for each treatment. Letters denote significant differences, ANOVA, d.f. = 5,24 F = 67.4460, p<0.001; Tukey HSD, p<0.05.
(EPS)

S3 Fig Comparison of DWV, IAPV, and SBV g.e. in *M*. *rotundata* and *C*. *inaequalis* samples collected at 1 and 5 days post treatment exposure (d.). ANOVA, Tukey HSD p<0.05 (S5 Table); sample sizes indicated, * denotes significantly different from field-collected honey bees, ⁺ denotes significantly different from apiary-collected honey bee samples.
(EPS)

S1 Table List of site names and descriptions for field sites used for bee collections with latitude and longitude and percent of total bees collected that were honey bees. (DOCX)

S2 Table Summary table of all the wild bees collected across field sites: a) List of each specimen collected, with family, genus, and species noted, as well as whether specimen was used for virus quantification. b) Counts and proportion detected for each virus by genus and family.
(DOCX)

S3 Table χ^2^ report statistics for virus incidence in each wild bee bee family compared to a) field-collected honey bees and b) apiary samples. Data used to generate Fig 1A.
(DOCX)

S4 Table Statistical reporting for mixed-model ANOVA and Tukey HSD posthoc tests comparing virus genome equivalents in wild bees to field-collected honey bees and apiary samples. Data used to generate Fig 1B.
(DOCX)

S5 Table χ^2^ report statistics comparing survival between control- and virus-treated *M*. *rotundata* and *C*. *inaequalis*. Used to generate Fig 2A.
(DOCX)

S6 Table Statistical reporting for mixed-model ANOVA and Tukey HSD posthoc tests comparing virus genome equivalents in *M*. *rotundata* and *C*. *inaequalis* compared to a) field-collected honey bees and b) apiary samples. Statistical reporting for mixed-model ANOVA and Tukey HSD posthoc tests comparing virus genome equivalents in wild bees compared to field-collected honey bees and apiary samples for a) *M*. *rotundata* and b) *C*. *inaequalis*.
(DOCX)
