# Supplementary figures and images for: Honey Bee Viruses in Wild Bees: Viral Prevalence, Loads, and Experimental Inoculation
Source: PLoS One. 2016 Nov 10;11(11):e0166190. doi: 10.1371/journal.pone.0166190 (PMC5104440; doi:10.1371/journal.pone.0166190)

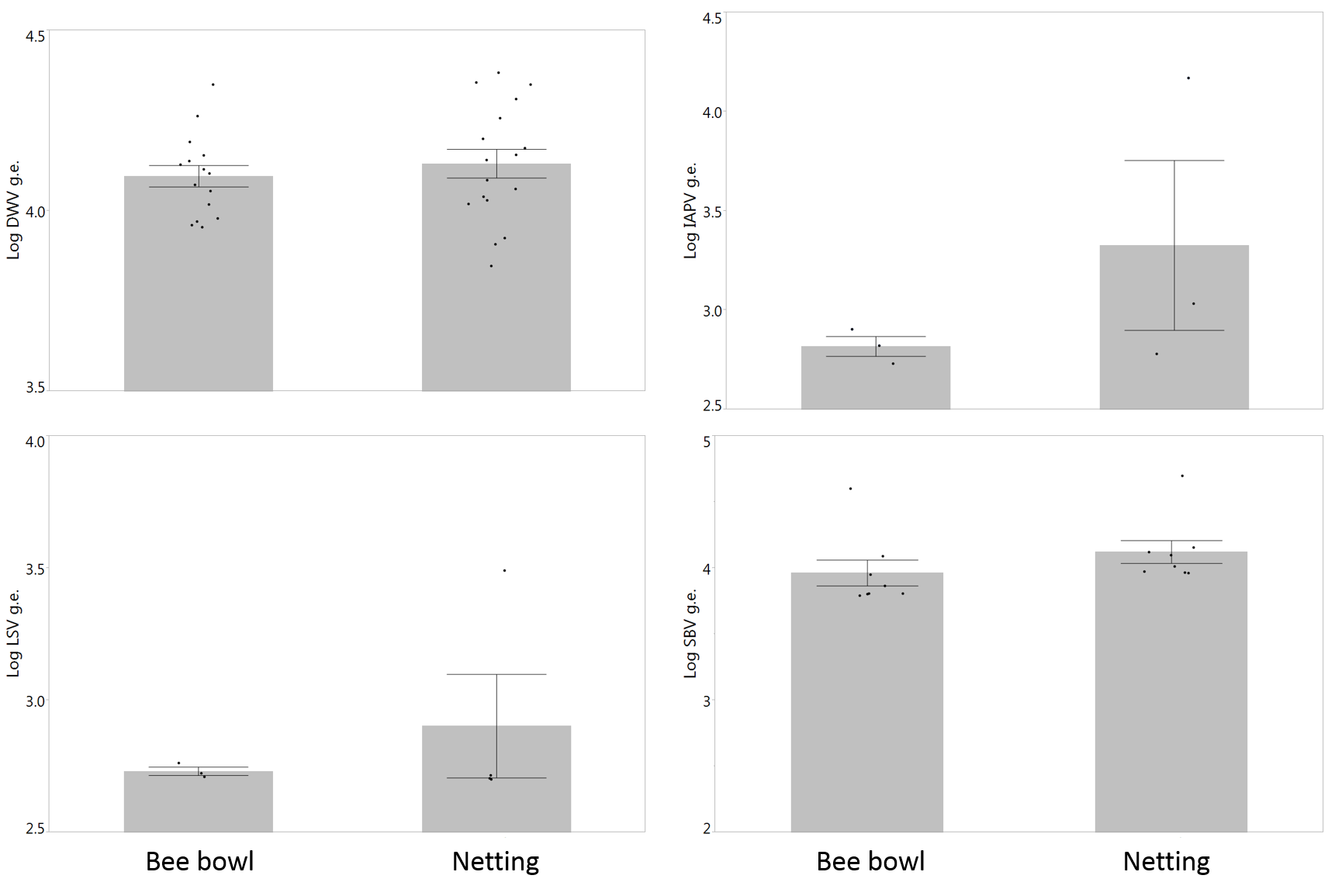

Supplement: S1 Fig — BQCV was too rarely detected for comparisons. Virus titers were not significantly different between any groups (t-test, p<0.05). This suggests that, even though samples had slightly different treatments due to collection methods, there was no significant effect on virus titer because of this. (TIF) [file pone.0166190.s001.tif]
