# Supplementary material for: Honey Bee Viruses in Wild Bees: Viral Prevalence, Loads, and Experimental Inoculation
Source: PLoS One. 2016 Nov 10;11(11):e0166190. doi: 10.1371/journal.pone.0166190 (PMC5104440; doi:10.1371/journal.pone.0166190)
Supplement: S1 Table — (DOCX) [file pone.0166190.s004.docx]

S1 Table: List of site names and descriptions for bee collections with latitude and longitude and percent of total bees collected that were honey bees.

| **Site Name** | **Description** | **County** | **Latitude** | **Longitude** | **total bees collected** | **Total honey bees** | **% honey bees** |
| --- | --- | --- | --- | --- | --- | --- | --- |
| Cayler Prairie Preserve | Remnant Prairie | Dickinson | 43.39601 | -95.244589 | 54 | 0 | 0 |
| Anderson Prairie State Preserve | Remnant Prairie | Emmet | 43.440093 | -94.875042 | 27 | 0 | 0 |
| Doolittle Prairie State Preserve | Remnant Prairie | Story | 42.148873 | -93.589461 | 40 | 8 | 20 |
| Neal Smith Wildlife Refuge | Restored Tallgrass Prairie | Jasper | 41.558752 | -93.267885 | 66 | 13 | 19.7 |
| Story County Soybean Field | Agriculture | Story | 41.982191 | -93.643938 | 97 | 3 | 3.01 |
