# Supplementary material for: Honey Bee Viruses in Wild Bees: Viral Prevalence, Loads, and Experimental Inoculation
Source: PLoS One. 2016 Nov 10;11(11):e0166190. doi: 10.1371/journal.pone.0166190 (PMC5104440; doi:10.1371/journal.pone.0166190)
Supplement: S2 Table — Summary table of all the wild bees collected across field sites: a) List of each specimen collected, with family, genus, and species noted, as well as whether specimen was used for virus quantification. b) Counts and proportion detected for each virus by genus and family. (DOCX) [file pone.0166190.s005.docx]

S2 Table a) List and identification of each specimen collected and whether it was used for virus quantification (1= yes; 0 =no)

| Sample code | *Genus* | *Species* | Family | Sample Site | Used for virus quantification |
| --- | --- | --- | --- | --- | --- |
| L68 | *Andrena* | *helianthiformis* | Andrenidae | Anderson prairie | 1 |
| L69 | *Andrena* | *helianthiformis* | Andrenidae | Anderson prairie | 1 |
| L70 | *Andrena* | *helianthiformis* | Andrenidae | Anderson prairie | 1 |
| L71 | *Andrena* | *helianthiformis* | Andrenidae | Anderson prairie | 1 |
| L72 | *Andrena* | *helianthiformis* | Andrenidae | Anderson prairie | 1 |
| L73 | *Andrena* | *helianthiformis* | Andrenidae | Anderson prairie | 1 |
| L74 | *Andrena* | *helianthiformis* | Andrenidae | Anderson prairie | 1 |
| L75 | *Andrena* | *helianthiformis* | Andrenidae | Anderson prairie | 1 |
| L76 | *Andrena* | *helianthiformis* | Andrenidae | Anderson prairie | 1 |
| L77 | *Andrena* | *helianthiformis* | Andrenidae | Anderson prairie | 0 |
| L78 | *Andrena* | *helianthiformis* | Andrenidae | Anderson prairie | 0 |
| L79 | *Andrena* | *helianthiformis* | Andrenidae | Anderson prairie | 0 |
| LP2 | *Andrena* | *helianthi* | Andrenidae | Anderson prairie | 1 |
| LP1 | *Andrena* | *helianthi* | Andrenidae | Calyer prairie | 1 |
| LP3 | *Andrena* | *wilkella* | Andrenidae | Calyer prairie | 1 |
| L42 | *Andrena* | *wilkella* | Andrenidae | Cayler prairie | 1 |
| L43 | *Andrena* | *wilkella* | Andrenidae | Cayler prairie | 1 |
| L44a | *Andrena* | *wilkella* | Andrenidae | Cayler prairie | 1 |
| L44b | *Halictus* | *rubicundus* | Andrenidae | Cayler prairie | 1 |
| L45 | *Andrena* | *wilkella* | Andrenidae | Cayler prairie | 1 |
| L46 | *Andrena* | *wilkella* | Andrenidae | Cayler prairie | 1 |
| L47 | *Andrena* | *wilkella* | Andrenidae | Cayler prairie | 1 |
| L48 | *Andrena* | *wilkella* | Andrenidae | Cayler prairie | 1 |
| L49 | *Andrena* | *wilkella* | Andrenidae | Cayler prairie | 1 |
| L50 | *Andrena* | *wilkella* | Andrenidae | Cayler prairie | 1 |
| L51 | *Andrena* | *wilkella* | Andrenidae | Cayler prairie | 1 |
| L52 | *Andrena* | *wilkella* | Andrenidae | Cayler prairie | 0 |
| L53 | *Andrena* | *wilkella* | Andrenidae | Cayler prairie | 0 |
| L54 | *Andrena* | *helianthiformis* | Andrenidae | Cayler prairie | 0 |
| L55 | *Andrena* | *helianthiformis* | Andrenidae | Cayler prairie | 0 |
| L56 | *Andrena* | *helianthiformis* | Andrenidae | Cayler prairie | 0 |
| L57 | *Andrena* | *helianthiformis* | Andrenidae | Cayler prairie | 1 |
| L58 | *Andrena* | *helianthiformis* | Andrenidae | Cayler prairie | 1 |
| L59 | *Andrena* | *helianthiformis* | Andrenidae | Cayler prairie | 1 |
| L60 | *Andrena* | *helianthiformis* | Andrenidae | Cayler prairie | 1 |
| L61 | *Andrena* | *helianthiformis* | Andrenidae | Cayler prairie | 1 |
| L62 | *Andrena* | *helianthiformis* | Andrenidae | Cayler prairie | 1 |
| L63 | *Andrena* | *helianthiformis* | Andrenidae | Cayler prairie | 1 |
| L64 | *Andrena* | *helianthiformis* | Andrenidae | Cayler prairie | 1 |
| L65 | *Andrena* | *helianthiformis* | Andrenidae | Cayler prairie | 1 |
| L66 | *Andrena* | *helianthiformis* | Andrenidae | Cayler prairie | 0 |
| L67 | *Andrena* | *helianthiformis* | Andrenidae | Cayler prairie | 0 |
| H13 | *Andrena* | *rudbeckiae* | Andrenidae | Neal Smith WLR | 1 |
| H14 | *Andrena* | *rudbeckiae* | Andrenidae | Neal Smith WLR | 1 |
| H15 | *Andrena* | *rudbeckiae* | Andrenidae | Neal Smith WLR | 1 |
| H16 | *Andrena* | *rudbeckiae* | Andrenidae | Neal Smith WLR | 1 |
| H17 | *Andrena* | *rudbeckiae* | Andrenidae | Neal Smith WLR | 1 |
| H18 | *Andrena* | *rudbeckiae* | Andrenidae | Neal Smith WLR | 1 |
| H19 | *Andrena* | *rudbeckiae* | Andrenidae | Neal Smith WLR | 1 |
| H70 | *Andrena* | *spp.* | Andrenidae | Neal Smith WLR | 0 |
| H71 | *Andrena* | *spp.* | Andrenidae | Neal Smith WLR | 0 |
| H72 | *Andrena* | *spp.* | Andrenidae | Neal Smith WLR | 0 |
| H73 | *Andrena* | *spp.* | Andrenidae | Neal Smith WLR | 0 |
| N52 | *Andrena* | *spp.* | Andrenidae | Soybean field | 0 |
| L82 | *Melissodes* | *bimaculata* | Apidae | Calyer prairie | 1 |
| D1 | *Apis* | *mellifera* | Apidae | Doolittle prairie | 1 |
| D15 | *Bombus* | *griseocollis* | Apidae | Doolittle prairie | 0 |
| D16 | *Bombus* | *griseocollis* | Apidae | Doolittle prairie | 1 |
| D17 | *Bombus* | *griseocollis* | Apidae | Doolittle prairie | 1 |
| D18 | *Bombus* | *griseocollis* | Apidae | Doolittle prairie | 1 |
| D19 | *Bombus* | *griseocollis* | Apidae | Doolittle prairie | 1 |
| D2 | *Apis* | *mellifera* | Apidae | Doolittle prairie | 1 |
| D20 | *Bombus* | *auricomus* | Apidae | Doolittle prairie | 1 |
| D21 | *Bombus* | *griseocollis* | Apidae | Doolittle prairie | 1 |
| D22 | *Bombus* | *griseocollis* | Apidae | Doolittle prairie | 1 |
| D23 | *Bombus* | *griseocollis* | Apidae | Doolittle prairie | 1 |
| D24 | *Bombus* | *auricomus* | Apidae | Doolittle prairie | 1 |
| D25 | *Bombus* | *auricomus* | Apidae | Doolittle prairie | 1 |
| D26 | *Bombus* | *pensylvanicus* | Apidae | Doolittle prairie | 1 |
| D27 | *Bombus* | *griseocollis* | Apidae | Doolittle prairie | 1 |
| D28 | *Bombus* | *griseocollis* | Apidae | Doolittle prairie | 1 |
| D29 | *Bombus* | *griseocollis* | Apidae | Doolittle prairie | 1 |
| D3 | *Apis* | *mellifera* | Apidae | Doolittle prairie | 1 |
| D30 | *Bombus* | *griseocollis* | Apidae | Doolittle prairie | 1 |
| D31 | *Bombus* | *griseocollis* | Apidae | Doolittle prairie | 1 |
| D32 | *Bombus* | *griseocollis* | Apidae | Doolittle prairie | 1 |
| D33 | *Bombus* | *griseocollis* | Apidae | Doolittle prairie | 1 |
| D34 | *Bombus* | *griseocollis* | Apidae | Doolittle prairie | 1 |
| D35 | *Bombus* | *auricomus* | Apidae | Doolittle prairie | 1 |
| D36 | *Bombus* | *pensylvanicus* | Apidae | Doolittle prairie | 1 |
| D37 | *Apis* | *mellifera* | Apidae | Doolittle prairie | 1 |
| D38 | *Apis* | *mellifera* | Apidae | Doolittle prairie | 1 |
| D39 | *Apis* | *mellifera* | Apidae | Doolittle prairie | 1 |
| D4 | *Apis* | *mellifera* | Apidae | Doolittle prairie | 1 |
| D40 | *Apis* | *mellifera* | Apidae | Doolittle prairie | 1 |
| H1 | *Apis* | *mellifera* | Apidae | Neal Smith WLR | 0 |
| H10 | *Apis* | *mellifera* | Apidae | Neal Smith WLR | 1 |
| H11 | *Apis* | *mellifera* | Apidae | Neal Smith WLR | 1 |
| H12 | *Apis* | *mellifera* | Apidae | Neal Smith WLR | 1 |
| H2 | *Apis* | *mellifera* | Apidae | Neal Smith WLR | 0 |
| H20 | *Bombus* | *griseocollis* | Apidae | Neal Smith WLR | 1 |
| H21 | *Bombus* | *griseocollis* | Apidae | Neal Smith WLR | 1 |
| H22 | *Bombus* | *griseocollis* | Apidae | Neal Smith WLR | 1 |
| H23 | *Bombus* | *griseocollis* | Apidae | Neal Smith WLR | 0 |
| H24 | *Bombus* | *griseocollis* | Apidae | Neal Smith WLR | 1 |
| H25 | *Bombus* | *griseocollis* | Apidae | Neal Smith WLR | 1 |
| H26 | *Bombus* | *griseocollis* | Apidae | Neal Smith WLR | 1 |
| H27 | *Bombus* | *griseocollis* | Apidae | Neal Smith WLR | 1 |
| H28 | *Bombus* | *griseocollis* | Apidae | Neal Smith WLR | 1 |
| H3 | *Apis* | *mellifera* | Apidae | Neal Smith WLR | 1 |
| H39 | *Apis* | *mellifera* | Apidae | Neal Smith WLR | 1 |
| H4 | *Apis* | *mellifera* | Apidae | Neal Smith WLR | 1 |
| H40 | *Bombus* | *occidentalis* | Apidae | Neal Smith WLR | 1 |
| H48 | *Ceratina* | *calcarata* | Apidae | Neal Smith WLR | 0 |
| H5 | *Apis* | *mellifera* | Apidae | Neal Smith WLR | 1 |
| H6 | *Apis* | *mellifera* | Apidae | Neal Smith WLR | 1 |
| H68 | *Melissodes* | *bimaculata* | Apidae | Neal Smith WLR | 0 |
| H69 | *Melissodes* | *bimaculata* | Apidae | Neal Smith WLR | 0 |
| H7 | *Apis* | *mellifera* | Apidae | Neal Smith WLR | 1 |
| H8 | *Apis* | *mellifera* | Apidae | Neal Smith WLR | 1 |
| H9 | *Apis* | *mellifera* | Apidae | Neal Smith WLR | 1 |
| N31 | *Bombus* | *griseocollis* | Apidae | Soybean field | 0 |
| N51 | *Apis* | *mellifera* | Apidae | Soybean field | 0 |
| N67 | *Apis* | *mellifera* | Apidae | Soybean field | 1 |
| N68 | *Apis* | *mellifera* | Apidae | Soybean field | 1 |
| N73 | *Bombus* | *impatiens* | Apidae | Soybean field | 1 |
| N74 | *Bombus* | *griseocollis* | Apidae | Soybean field | 1 |
| N75 | *Bombus* | *auricomus* | Apidae | Soybean field | 1 |
| N81 | *Melissodes* | *bimaculata* | Apidae | Soybean field | 1 |
| N82 | *Melissodes* | *bimaculata* | Apidae | Soybean field | 0 |
| N83 | *Melissodes* | *bimaculata* | Apidae | Soybean field | 1 |
| N84 | *Melissodes* | *bimaculata* | Apidae | Soybean field | 1 |
| N85 | *Melissodes* | *bimaculata* | Apidae | Soybean field | 1 |
| N86 | *Melissodes* | *bimaculata* | Apidae | Soybean field | 1 |
| N87 | *Melissodes* | *bimaculata* | Apidae | Soybean field | 1 |
| N88 | *Melissodes* | *bimaculata* | Apidae | Soybean field | 1 |
| N89 | *Melissodes* | *bimaculata* | Apidae | Soybean field | 1 |
| N90 | *Melissodes* | *bimaculata* | Apidae | Soybean field | 1 |
| N91 | *Melissodes* | *bimaculata* | Apidae | Soybean field | 0 |
| N92 | *Melissodes* | *bimaculata* | Apidae | Soybean field | 0 |
| N93 | *Melissodes* | *bimaculata* | Apidae | Soybean field | 0 |
| N94 | *Melissodes* | *bimaculata* | Apidae | Soybean field | 0 |
| N95 | *Melissodes* | *bimaculata* | Apidae | Soybean field | 0 |
| NP2 | *Melissodes* | *bimaculata* | Apidae | Soybean field | 1 |
| H43 | *Hylaeus* | *punctatus* | Colletidae | Neal Smith WLR | 0 |
| H44 | *Hylaeus* | *punctatus* | Colletidae | Neal Smith WLR | 0 |
| H45 | *Hylaeus* | *punctatus* | Colletidae | Neal Smith WLR | 0 |
| L24 | *Halictus* | *ligatus* | Halictidae | Anderson prairie | 0 |
| L25 | *Halictus* | *ligatus* | Halictidae | Anderson prairie | 1 |
| L26 | *Halictus* | *ligatus* | Halictidae | Anderson prairie | 0 |
| L27 | *Halictus* | *rubicundus* | Halictidae | Anderson prairie | 0 |
| L28 | *Halictus* | *rubicundus* | Halictidae | Anderson prairie | 0 |
| L29 | *Halictus* | *rubicundus* | Halictidae | Anderson prairie | 0 |
| L30 | *Halictus* | *rubicundus* | Halictidae | Anderson prairie | 0 |
| L31 | *Halictus* | *rubicundus* | Halictidae | Anderson prairie | 0 |
| L32 | *Halictus* | *rubicundus* | Halictidae | Anderson prairie | 0 |
| L33 | *Halictus* | *rubicundus* | Halictidae | Anderson prairie | 0 |
| L38 | *Lasioglossum* | *Dialectus* | Halictidae | Anderson prairie | 0 |
| L39 | *Lasioglossum* | *Dialectus* | Halictidae | Anderson prairie | 0 |
| L40 | *Lasioglossum* | *Dialectus* | Halictidae | Anderson prairie | 0 |
| L41 | *Lasioglossum* | *Dialectus* | Halictidae | Anderson prairie | 0 |
| L1 | *Halictus* | *ligatus* | Halictidae | Calyer prairie | 1 |
| L11 | *Halictus* | *ligatus* | Halictidae | Calyer prairie | 1 |
| L12 | *Halictus* | *ligatus* | Halictidae | Calyer prairie | 1 |
| L13 | *Halictus* | *ligatus* | Halictidae | Calyer prairie | 1 |
| L14 | *Halictus* | *ligatus* | Halictidae | Calyer prairie | 1 |
| L15 | *Halictus* | *ligatus* | Halictidae | Calyer prairie | 1 |
| L16 | *Halictus* | *ligatus* | Halictidae | Calyer prairie | 1 |
| L17 | *Halictus* | *ligatus* | Halictidae | Calyer prairie | 0 |
| L18 | *Halictus* | *ligatus* | Halictidae | Calyer prairie | 0 |
| L19 | *Halictus* | *ligatus* | Halictidae | Calyer prairie | 0 |
| L2 | *Halictus* | *rubicundus* | Halictidae | Calyer prairie | 1 |
| L20 | *Halictus* | *ligatus* | Halictidae | Calyer prairie | 0 |
| L21 | *Halictus* | *rubicundus* | Halictidae | Calyer prairie | 0 |
| L22 | *Agapostemon* | *viriscens* | Halictidae | Calyer prairie | 0 |
| L23 | *Augochlorella* | *auratus* | Halictidae | Calyer prairie | 0 |
| L3 | *Halictus* | *rubicundus* | Halictidae | Calyer prairie | 1 |
| L34 | *Agapostemon* | *viriscens* | Halictidae | Calyer prairie | 0 |
| L4 | *Halictus* | *ligatus* | Halictidae | Calyer prairie | 1 |
| L5 | *Halictus* | *ligatus* | Halictidae | Calyer prairie | 1 |
| L6 | *Halictus* | *rubicundus* | Halictidae | Calyer prairie | 1 |
| L7 | *Halictus* | *rubicundus* | Halictidae | Calyer prairie | 1 |
| L8 | *Halictus* | *rubicundus* | Halictidae | Calyer prairie | 1 |
| L9 | *Halictus* | *rubicundus* | Halictidae | Calyer prairie | 1 |
| LP4 | *Halictus* | *ligatus* | Halictidae | Calyer prairie | 1 |
| D12 | *Augochlorella* | *auratus* | Halictidae | Doolittle prairie | 1 |
| D13 | *Augochlorella* | *auratus* | Halictidae | Doolittle prairie | 1 |
| D14 | *Augochlora* | *pura* | Halictidae | Doolittle prairie | 0 |
| H34 | *Halictus* | *parallelus* | Halictidae | Neal Smith WLR | 1 |
| H35 | *Halictus* | *parallelus* | Halictidae | Neal Smith WLR | 1 |
| H36 | *Halictus* | *parallelus* | Halictidae | Neal Smith WLR | 1 |
| H37 | *Halictus* | *parallelus* | Halictidae | Neal Smith WLR | 1 |
| H38 | *Halictus* | *parallelus* | Halictidae | Neal Smith WLR | 1 |
| H41 | *Agapostemon* | *viriscens* | Halictidae | Neal Smith WLR | 0 |
| H42 | *Agapostemon* | *viriscens* | Halictidae | Neal Smith WLR | 0 |
| H46 | *Lasioglossum* | *spp* | Halictidae | Neal Smith WLR | 0 |
| H47 | *Lasioglossum* | *spp* | Halictidae | Neal Smith WLR | 0 |
| H62 | *Augochlorella* | *auratus* | Halictidae | Neal Smith WLR | 0 |
| H63 | *Augochlorella* | *auratus* | Halictidae | Neal Smith WLR | 0 |
| H64 | *Augochlorella* | *auratus* | Halictidae | Neal Smith WLR | 0 |
| H65 | *Augochlorella* | *auratus* | Halictidae | Neal Smith WLR | 1 |
| H66 | *Augochlorella* | *auratus* | Halictidae | Neal Smith WLR | 0 |
| H67 | *Augochlorella* | *auratus* | Halictidae | Neal Smith WLR | 1 |
| N43 | *Halictus* | *rubicundus* | Halictidae | Soybean field | 1 |
| N1 | *Agapostemon* | *viriscens* | Halictidae | Soybean field | 0 |
| N10 | *Agapostemon* | *viriscens* | Halictidae | Soybean field | 1 |
| N11 | *Agapostemon* | *viriscens* | Halictidae | Soybean field | 1 |
| N12 | *Agapostemon* | *viriscens* | Halictidae | Soybean field | 1 |
| N13 | *Agapostemon* | *viriscens* | Halictidae | Soybean field | 1 |
| N14 | *Agapostemon* | *viriscens* | Halictidae | Soybean field | 0 |
| N15 | *Agapostemon* | *viriscens* | Halictidae | Soybean field | 0 |
| N16 | *Agapostemon* | *viriscens* | Halictidae | Soybean field | 0 |
| N17 | *Agapostemon* | *viriscens* | Halictidae | Soybean field | 0 |
| N18 | *Agapostemon* | *viriscens* | Halictidae | Soybean field | 0 |
| N19 | *Augochlorella* | *auratus* | Halictidae | Soybean field | 0 |
| N2 | *Agapostemon* | *viriscens* | Halictidae | Soybean field | 1 |
| N20 | *Agapostemon* | *viriscens* | Halictidae | Soybean field | 0 |
| N21 | *Agapostemon* | *viriscens* | Halictidae | Soybean field | 0 |
| N22 | *Agapostemon* | *viriscens* | Halictidae | Soybean field | 0 |
| N23 | *Agapostemon* | *viriscens* | Halictidae | Soybean field | 0 |
| N24 | *Agapostemon* | *viriscens* | Halictidae | Soybean field | 0 |
| N25 | *Agapostemon* | *viriscens* | Halictidae | Soybean field | 0 |
| N26 | *Halictus* | *confusus* | Halictidae | Soybean field | 0 |
| N27 | *Halictus* | *confusus* | Halictidae | Soybean field | 0 |
| N28 | *Augochlorella* | *auratus* | Halictidae | Soybean field | 0 |
| N29 | *Halictus* | *confusus* | Halictidae | Soybean field | 0 |
| N3 | *Agapostemon* | *viriscens* | Halictidae | Soybean field | 1 |
| N30 | *Augochlorella* | *auratus* | Halictidae | Soybean field | 0 |
| N32 | *Halictus* | *confusus* | Halictidae | Soybean field | 0 |
| N33 | *Augochlorella* | *auratus* | Halictidae | Soybean field | 0 |
| N34 | *Augochlorella* | *auratus* | Halictidae | Soybean field | 0 |
| N35 | *Halictus* | *confusus* | Halictidae | Soybean field | 0 |
| N36 | *Augochlorella* | *auratus* | Halictidae | Soybean field | 0 |
| N37 | *Halictus* | *rubicundus* | Halictidae | Soybean field | 0 |
| N38 | *Halictus* | *rubicundus* | Halictidae | Soybean field | 1 |
| N39 | *Halictus* | *rubicundus* | Halictidae | Soybean field | 1 |
| N4 | *Agapostemon* | *viriscens* | Halictidae | Soybean field | 1 |
| N40 | *Halictus* | *rubicundus* | Halictidae | Soybean field | 0 |
| N41 | *Halictus* | *rubicundus* | Halictidae | Soybean field | 1 |
| N42 | *Halictus* | *rubicundus* | Halictidae | Soybean field | 1 |
| N44 | *Halictus* | *rubicundus* | Halictidae | Soybean field | 1 |
| N45 | *Halictus* | *ligatus* | Halictidae | Soybean field | 1 |
| N46 | *Halictus* | *ligatus* | Halictidae | Soybean field | 1 |
| N47 | *Halictus* | *ligatus* | Halictidae | Soybean field | 1 |
| N48 | *Halictus* | *ligatus* | Halictidae | Soybean field | 0 |
| N49 | *Halictus* | *ligatus* | Halictidae | Soybean field | 0 |
| N5 | *Agapostemon* | *viriscens* | Halictidae | Soybean field | 1 |
| N50 | *Halictus* | *ligatus* | Halictidae | Soybean field | 0 |
| N53 | *Lasioglossumsubg.* | *Dialectus* | Halictidae | Soybean field | 0 |
| N54 | *Lasioglossumsubg.* | *Dialectus* | Halictidae | Soybean field | 0 |
| N55 | *Lasioglossumsubg.* | *Dialectus* | Halictidae | Soybean field | 0 |
| N56 | *Lasioglossumsubg.* | *Dialectus* | Halictidae | Soybean field | 0 |
| N57 | *Lasioglossumsubg.* | *Dialectus* | Halictidae | Soybean field | 0 |
| N58 | *Lasioglossumsubg.* | *Dialectus* | Halictidae | Soybean field | 0 |
| N59 | *Lasioglossumsubg.* | *Dialectus* | Halictidae | Soybean field | 0 |
| N6 | *Agapostemon* | *viriscens* | Halictidae | Soybean field | 1 |
| N60 | *Lasioglossumsubg.* | *Dialectus* | Halictidae | Soybean field | 0 |
| N61 | *Lasioglossumsubg.* | *Dialectus* | Halictidae | Soybean field | 0 |
| N62 | *Lasioglossumsubg.* | *Dialectus* | Halictidae | Soybean field | 0 |
| N63 | *Lasioglossumsubg.* | *Dialectus* | Halictidae | Soybean field | 0 |
| N64 | *Lasioglossumsubg.* | *Dialectus* | Halictidae | Soybean field | 0 |
| N65 | *Lasioglossumsubg.* | *Dialectus* | Halictidae | Soybean field | 0 |
| N66 | *Lasioglossumsubg.* | *Dialectus* | Halictidae | Soybean field | 0 |
| N69 | *Augochlorella* | *auratus* | Halictidae | Soybean field | 1 |
| N7 | *Agapostemon* | *viriscens* | Halictidae | Soybean field | 0 |
| N70 | *Augochlorella* | *auratus* | Halictidae | Soybean field | 0 |
| N71 | *Augochlorella* | *auratus* | Halictidae | Soybean field | 1 |
| N72 | *Augochlorella* | *auratus* | Halictidae | Soybean field | 0 |
| N8 | *Agapostemon* | *viriscens* | Halictidae | Soybean field | 1 |
| N9 | *Agapostemon* | *viriscens* | Halictidae | Soybean field | 1 |
| NP1 | *Agapostemon* | *viriscens* | Halictidae | Soybean field | 1 |
| D10 | *Megachile* | *brevis* | Megachilidae | Doolittle prairie | 1 |
| D11 | *Megachile* | *brevis* | Megachilidae | Doolittle prairie | 1 |
| D5 | *Megachile* | *brevis* | Megachilidae | Doolittle prairie | 1 |
| D6 | *Megachile* | *brevis* | Megachilidae | Doolittle prairie | 1 |
| D7 | *Megachile* | *brevis* | Megachilidae | Doolittle prairie | 1 |
| D8 | *Megachile* | *brevis* | Megachilidae | Doolittle prairie | 1 |
| D9 | *Megachile* | *brevis* | Megachilidae | Doolittle prairie | 1 |
| H29 | *Megachile* | *centuncularis* | Megachilidae | Neal Smith WLR | 0 |
| H30 | *Megachile* | *brevis* | Megachilidae | Neal Smith WLR | 1 |
| H31 | *Megachile* | *brevis* | Megachilidae | Neal Smith WLR | 1 |
| H32 | *Megachile* | *xylocopoides* | Megachilidae | Neal Smith WLR | 0 |
| H33 | *Megachile* | *xylocopoides* | Megachilidae | Neal Smith WLR | 0 |
| H74 | *Megachile* | *xylocopoides* | Megachilidae | Neal Smith WLR | 0 |
| H75 | *Megachile* | *xylocopoides* | Megachilidae | Neal Smith WLR | 0 |
| H76 | *Megachile* | *brevis* | Megachilidae | Neal Smith WLR | 1 |
| H77 | *Megachile* | *brevis* | Megachilidae | Neal Smith WLR | 1 |
| H78 | *Megachile* | *brevis* | Megachilidae | Neal Smith WLR | 1 |
| H79 | *Megachile* | *brevis* | Megachilidae | Neal Smith WLR | 0 |
| N76 | *Megachile* | *brevis* | Megachilidae | Soybean field | 1 |
| N77 | *Megachile* | *brevis* | Megachilidae | Soybean field | 0 |
| N78 | *Megachile* | *brevis* | Megachilidae | Soybean field | 1 |
| N79 | *Megachile* | *brevis* | Megachilidae | Soybean field | 1 |
| N80 | *Megachile* | *brevis* | Megachilidae | Soybean field | 1 |

S2 Table b) Sample size, count, and proportion detection for each tested virus by genus and family

| **Genus** | N | With DWV detection | With BQCV detection | With IAPV detection | With LSV detection | With SBV detection | DWV prop. | BQCV prop. | IAPV prop. | LSV prop. | SBV prop. |
| --- | --- | --- | --- | --- | --- | --- | --- | --- | --- | --- | --- |
| Agapostemon | 12 | 8 | 0 | 2 | 3 | 5 | 0.666667 | 0 | 0.166667 | 0.25 | 0.416667 |
| Andrena | 38 | 18 | 0 | 21 | 2 | 14 | 0.473684 | 0 | 0.552632 | 0.052632 | 0.368421 |
| Apiary hive | 25 | 11 | 25 | 7 | 16 | 23 | 0.44 | 1 | 0.28 | 0.64 | 0.92 |
| Apis | 21 | 11 | 18 | 1 | 11 | 15 | 0.52381 | 0.857143 | 0.047619 | 0.52381 | 0.714286 |
| Augochlorella | 6 | 5 | 1 | 0 | 2 | 6 | 0.833333 | 0.166667 | 0 | 0.333333 | 1 |
| Bombus | 33 | 15 | 2 | 4 | 5 | 17 | 0.454545 | 0.060606 | 0.121212 | 0.151515 | 0.515152 |
| Halictus | 32 | 20 | 1 | 1 | 2 | 5 | 0.625 | 0.03125 | 0.03125 | 0.0625 | 0.15625 |
| Megachile | 17 | 9 | 0 | 2 | 3 | 12 | 0.529412 | 0 | 0.117647 | 0.176471 | 0.705882 |
| Melissodes | 10 | 6 | 0 | 2 | 1 | 8 | 0.6 | 0 | 0.2 | 0.1 | 0.8 |
| total | 194 | 103 | 47 | 40 | 45 | 105 | 0.530928 | 0.242268 | 0.206186 | 0.231959 | 0.541237 |
|  |  |  |  |  |  |  |  |  |  |  |  |
|  | | |  |  |  |  |  |  |  |  |  |
| **Family** |  |  |  |  |  |  |  |  |  |  |  |
| Andrenidae | 38 | 18 | 0 | 21 | 2 | 14 | 0.473684 | 0 | 0.552632 | 0.052632 | 0.368421 |
| Apiary hive | 25 | 11 | 25 | 7 | 16 | 23 | 0.44 | 1 | 0.28 | 0.64 | 0.92 |
| Apidae (non-Apis) | 43 | 21 | 2 | 6 | 6 | 25 | 0.488372 | 0.046512 | 0.139535 | 0.139535 | 0.581395 |
| Field collected honey bee | 21 | 11 | 18 | 1 | 11 | 15 | 0.52381 | 0.857143 | 0.047619 | 0.52381 | 0.714286 |
| Halictidae | 50 | 33 | 2 | 3 | 7 | 16 | 0.66 | 0.04 | 0.06 | 0.14 | 0.32 |
| Megachilidae | 17 | 9 | 0 | 2 | 3 | 12 | 0.529412 | 0 | 0.117647 | 0.176471 | 0.705882 |
| total | 194 | 103 | 47 | 40 | 45 | 105 | 0.530928 | 0.242268 | 0.206186 | 0.231959 | 0.541237 |
