# Supplementary material for: Honey Bee Viruses in Wild Bees: Viral Prevalence, Loads, and Experimental Inoculation
Source: PLoS One. 2016 Nov 10;11(11):e0166190. doi: 10.1371/journal.pone.0166190 (PMC5104440; doi:10.1371/journal.pone.0166190)
Supplement: S3 Table — χ2 report statistics for virus incidence in each wild bee bee family compared to a) field-collected honey bees and b) apiary samples. Data used to generate Fig 1A. (DOCX) [file pone.0166190.s006.docx]

S3 Table: Chi squared statistics comparing virus incidence of wild bees to a) foraging honey bees and b) apiary-collected honey bees

|  | | | | | | | | | | | | |  |  |  |  |  |  |  |  |
| --- | --- | --- | --- | --- | --- | --- | --- | --- | --- | --- | --- | --- | --- | --- | --- | --- | --- | --- | --- | --- |
| a. Compared to Field-collected honey bees |  |  |  |  |  |  |  |  |  |  |  |  |  |  |  |  |  |  |  |  |
|  | BQCV |  |  |  | DWV |  |  |  | IAPV |  |  |  | LSV |  |  |  | SBV |  |  |  |
| Bee group | d.f. | N | χ² | p | d.f. | N | χ² | p | d.f. | N | χ² | p | d.f. | N | χ² | p | d.f. | N | χ² | p |
| Andrenidae | 1 | 59 | 55.358 | <0.0001 | 1 | 59 | 0.136 | 0.7123 | 1 | 59 | 17.638 | <0.0001 | 1 | 59 | 17.491 | <0.0001 | 1 | 59 | 6.631 | **0.01** |
| Apidae (non-Apis) | 1 | 64 | 46.096 | <0.0001 | 1 | 64 | 0.071 | 0.79 | 1 | 64 | 1.392 | 0.2381 | 1 | 64 | 10.275 | 0.0013 | 1 | 64 | 1.087 | 0.2972 |
| Halictidae | 1 | 71 | 50.406 | <0.0001 | 1 | 71 | 1.149 | 0.2838 |  |  | 0.044 | 0.8339 | 1 | 71 | 10.835 | 0.001 | 1 | 71 | 9.469 | 0.0021 |
| Megachilidae | 1 | 38 | 35.349 | <0.0001 | 1 | 38 | 0.001 | 0.9725 | 1 | 38 | 0.635 | 0.4256 | 1 | 38 | 5.108 | 0.0238 | 1 | 38 | 0.003 | 0.9547 |
|  |  |  |  |  |  |  |  |  |  |  |  |  |  |  |  |  |  |  |  |  |
| b. Compared to apiary-collected honey bees | | |  |  |  |  |  |  |  |  |  |  |  |  |  |  |  |  |  |  |
|  | BQCV |  |  |  | DWV |  |  |  | IAPV |  |  |  | LSV |  |  |  | SBV |  |  |  |
| Bee group | d.f. | N | χ² | p | d.f. | N | χ² | p | d.f. | N | χ² | p | d.f. | N | χ² | p | d.f. | N | χ² | p |
| Andrenidae | 1 | 64 | 77.158 | <0.0001 | 1 | 64 | 0.029 | 0.8658 | 1 | 64 | 4.238 | **0.0395** | 1 | 64 | 24.025 | <0.0001 | 1 | 64 | 22.295 | <0.0001 |
| Apidae (non-Apis) | 1 | 69 | 76.095 | <0.0001 | 1 | 69 | 0.089 | 0.7652 | 1 | 69 | 2.08 | 0.1492 | 1 | 69 | 18.665 | <0.0001 | 1 | 69 | 9.606 | 0.0019 |
| Halictidae | 1 | 80 | 85.115 | <0.0001 | 1 | 80 | 2.697 | 0.1005 | 1 | 80 | 7.35 | 0.0067 | 1 | 80 | 21.384 | <0.0001 | 1 | 80 | 27.37 | <0.0001 |
| Megachilidae | 1 | 42 | 56.691 | <0.0001 | 1 | 42 | 0.324 | 0.5689 | 1 | 42 | 1.682 | 0.1947 | 1 | 42 | 9.328 | 0.0023 | 1 | 42 | 3.312 | 0.0688 |
| Field *Apis mellifera* | 1 | 46 | 4.955 | 0.026 | 1 | 46 | 0.322 | 0.5707 | 1 | 46 | 4.819 | 0.0281 | 1 | 46 | 0.636 | 0.4253 | 1 | 46 | 3.362 | 0.0667 |
