# Supplementary material for: Honey Bee Viruses in Wild Bees: Viral Prevalence, Loads, and Experimental Inoculation
Source: PLoS One. 2016 Nov 10;11(11):e0166190. doi: 10.1371/journal.pone.0166190 (PMC5104440; doi:10.1371/journal.pone.0166190)
Supplement: S4 Table — Data used to generate Fig 1B. (DOCX) [file pone.0166190.s007.docx]

S4 Table: Statistical information for comparison of virus levels between wild bees and field-collected and apiary-collected honey bees

|  | |
| --- | --- |
|  |  |
| **BQCV comparisons** | |
| Mixed model ANOVA, df = 1,2; F=12.7186; p=0.0704 |  |
| Tukey HSD posthoc test | p-value |
| **Wild bees vs Field Honey bees** |  |
| Andrenidae | N/A |
| Apidae (non-Apis) | N/A |
| Halictidae | N/A |
| Megachilidae | N/A |
| **Wild bees vs Apiary** |  |
| Andrenidae | N/A |
| Apidae (non-Apis) | N/A |
| Halictidae | N/A |
| Megachilidae | N/A |
| **Field Honey bees vs Apiary** | N/A |
|  |  |
|  |  |
| **DWV comparisons** | |
| Mixed model ANOVA, df = 5, 91; F=20.931; p<0.0001 | |
| Tukey HSD posthoc test | p-value |
| **Wild bees vs Field Honey bees** |  |
| Andrenidae | 1 |
| Apidae (non-Apis) | 1 |
| Halictidae | 1 |
| Megachilidae | 0.998 |
| **Wild bees vs Apiary** |  |
| Andrenidae | <0.00001 |
| Apidae (non-Apis) | <0.00001 |
| Halictidae | <0.00001 |
| Megachilidae | <0.00001 |
| **Field Honey bees vs Apiary** | <0.00001 |
|  |  |
|  |  |
| **IAPV comparisons** | |
| Mixed model ANOVA, df = 2, 22; F=15.0055; p<0.0001 | |
| Tukey HSD posthoc test | p-value |
| **Wild bees vs Field Honey bees** |  |
| Andrenidae | 0.882 |
| Apidae (non-Apis) | N/A |
| Halictidae | N/A |
| Megachilidae | N/A |
| **Wild bees vs Apiary** |  |
| Andrenidae | <0.00001 |
| Apidae (non-Apis) | N/A |
| Halictidae | N/A |
| Megachilidae | N/A |
| **Field Honey bees vs Apiary** | <0.00001 |
|  |  |
|  |  |
| **LSV comparisons** | |
| Mixed model ANOVA, df = 3, 31; F=7.8109; p<0.0005 | |
| Tukey HSD posthoc test | p-value |
| **Wild bees vs Field Honey bees** |  |
| Andrenidae | N/A |
| Apidae (non-Apis) | 0.9942 |
| Halictidae | 0.6714 |
| Megachilidae | N/A |
| **Wild bees vs Apiary** |  |
| Andrenidae | N/A |
| Apidae (non-Apis) | 0.042 |
| Halictidae | <0.001 |
| Megachilidae | N/A |
| **Field Honey bees vs Apiary** | 0.0109 |
|  |  |
|  |  |
| **SBV comparisons** | |
| Mixed model ANOVA, df = 5, 91; F=2.28525; p=0.0526 | |
| Tukey HSD posthoc test | p-value |
| **Wild bees vs Field Honey bees** |  |
| Andrenidae | N/A |
| Apidae (non-Apis) | N/A |
| Halictidae | N/A |
| Megachilidae | N/A |
| **Wild bees vs Apiary** |  |
| Andrenidae | N/A |
| Apidae (non-Apis) | N/A |
| Halictidae | N/A |
| Megachilidae | N/A |
| **Field Honey bees vs Apiary** | N/A |
