# Supplementary material for: Honey Bee Viruses in Wild Bees: Viral Prevalence, Loads, and Experimental Inoculation
Source: PLoS One. 2016 Nov 10;11(11):e0166190. doi: 10.1371/journal.pone.0166190 (PMC5104440; doi:10.1371/journal.pone.0166190)
Supplement: S5 Table — Used to generate Fig 2A. (DOCX) [file pone.0166190.s008.docx]

S5 Table: χ² report statistics comparing survival between control- and virus-treated *M. rotundata* and *C. inaequalis*

| Morality of control- vs virus- treated solitary bees | | | | |
| --- | --- | --- | --- | --- |
| Species | d.f. | N | χ² | p |
| *M. rotundata* | 1 | 48 | 0.775 | 0.3787 |
| *C. inaequalis* | 1 | 63 | 2.688 | 0.1011 |
