# Supplementary material for: Honey Bee Viruses in Wild Bees: Viral Prevalence, Loads, and Experimental Inoculation
Source: PLoS One. 2016 Nov 10;11(11):e0166190. doi: 10.1371/journal.pone.0166190 (PMC5104440; doi:10.1371/journal.pone.0166190)
Supplement: S6 Table — Statistical reporting for mixed-model ANOVA and Tukey HSD posthoc tests comparing virus genome equivalents in M. rotundata and C. inaequalis compared to a) field-collected honey bees and b) apiary samples. Statistical reporting for mixed-model ANOVA and Tukey HSD posthoc tests comparing virus genome equivalents in wild bees compared to field-collected honey bees and apiary samples for a) M. rotundata and b) C. inaequalis. (DOCX) [file pone.0166190.s009.docx]

S6 Table: Statistical information for comparisons of treated *M. rotundata* and *C. inaequalis* to field-collected and apiary-collected honey bees

| a. Compared to Field-collected honey bees | | |  |  |  |  |  |  |  |  |  |  |  |  |  |  |
| --- | --- | --- | --- | --- | --- | --- | --- | --- | --- | --- | --- | --- | --- | --- | --- | --- |
|  | BQCV |  |  |  | DWV |  |  |  | IAPV |  |  |  | SBV |  |  |  |
| Species | d.f. | N | χ² | p | d.f. | N | χ² | p | d.f. | N | χ² | p | d.f. | N | χ² | p |
| *M. rotundata* | 1 | 41 | 39.002 | <0.0001 | 1 | 41 | 15.992 | <0.0001 | 1 | 41 | 48.773 | <0.0001 | 1 | 41 | 28.723 | <0.0001 |
| *C. inaequalis* | 1 | 41 | 26.585 | <0.0001 | 1 | 41 | 6.671 | 0.0116 | 1 | 41 | 48.779 | <0.0001 | 1 | 41 | 21.778 | <0.0001 |
|  |  |  |  |  |  |  |  |  |  |  |  |  |  |  |  |  |
| b. Compared to apiary-collected honey bees | | | |  |  |  |  |  |  |  |  |  |  |  |  |  |
|  | BQCV |  |  |  | DWV |  |  |  | IAPV |  |  |  | SBV |  |  |  |
| Species | d.f. | N | χ² | p | d.f. | N | χ² | p | d.f. | N | χ² | p | d.f. | N | χ² | p |
| *M. rotundata* | 1 | 45 | 61.827 | <0.0001 | 1 | 45 | 20.747 | <0.0001 | 1 | 45 | 30.923 | <0.0001 | 1 | 45 | 48.423 | <0.0001 |
| *C. inaequalis* | 1 | 45 | 47.568 | <0.0001 | 1 | 45 | 4.594 | 0.0321 | 1 | 45 | 30.923 | <0.0001 | 1 | 45 | 40.304 | <0.0001 |

| c. *M. rotundata* |  |
| --- | --- |
|  |  |
|  |  |
|  |  |
| **DWV comparisons** | |
| Mixed model ANOVA, df = 5,55; F=5.6204; p=0.00029 | |
| Tukey HSD posthoc test | p-value |
| **Treatments vs Field Honey bees** |  |
| Control, Day 1 | >0.05 |
| Control, Day 5 | >0.05 |
| Virus, Day 1 | >0.05 |
| Virus, Day 5 | >0.05 |
| **Treatments vs Apiary** |  |
| Control, Day 1 | >0.05 |
| Control, Day 5 | 0.0298 |
| Virus, Day 1 | >0.05 |
| Virus, Day 5 | 0.0474 |
|  |  |
|  |  |
| **IAPV comparisons** | |
| Mixed model ANOVA, df = 5, 40; F=10.128; p<0.0002 | |
| Tukey HSD posthoc test | p-value |
| **Treatments vs Field Honey bees** |  |
| Control, Day 1 | >0.05 |
| Control, Day 5 | >0.05 |
| Virus, Day 1 | >0.05 |
| Virus, Day 5 | >0.05 |
| **Treatments vs Apiary** |  |
| Control, Day 1 | <0.001 |
| Control, Day 5 | <0.001 |
| Virus, Day 1 | 0.00347 |
| Virus, Day 5 | <0.001 |
|  |  |
|  |  |
| **SBV comparisons** | |
| Mixed model ANOVA, df = 3, 49; F=6.3289; p=0.001029 | |
| Tukey HSD posthoc test | p-value |
| **Treatments vs Field Honey bees** |  |
| Control, Day 1 | N/A |
| Control, Day 5 | N/A |
| Virus, Day 1 | >0.05 |
| Virus, Day 5 | >0.05 |
| **Treatments vs Apiary** |  |
| Control, Day 1 | N/A |
| Control, Day 5 | N/A |
| Virus, Day 1 | 0.0368 |
| Virus, Day 5 | 0.03 |
|  |  |
|  |  |
|  |  |
| d. *C. inaequalis* |  |
|  |  |
|  |  |
|  |  |
|  |  |
| **DWV comparisons** | |
| Mixed model ANOVA, df = 4, 27; F=4.3657; p=0.007498 | |
| Tukey HSD posthoc test | p-value |
| **Treatments vs Field Honey bees** |  |
| Control, Day 1 | Control, Day 1 |
| Control, Day 5 | >0.05 |
| Virus, Day 1 | >0.05 |
| Virus, Day 5 | >0.05 |
| **Treatments vs Apiary** |  |
| Control, Day 1 | >0.05 |
| Control, Day 5 | >0.05 |
| Virus, Day 1 | >0.05 |
| Virus, Day 5 | >0.05 |
|  |  |
|  |  |
| **IAPV comparisons** | |
| Mixed model ANOVA, df = 5, 42; F=4.2433, p=0.003266 | |
| Tukey HSD posthoc test | p-value |
| **Treatments vs Field Honey bees** |  |
| Control, Day 1 | >0.05 |
| Control, Day 5 | >0.05 |
| Virus, Day 1 | >0.05 |
| Virus, Day 5 | >0.05 |
| **Treatments vs Apiary** |  |
| Control, Day 1 | 0.0341 |
| Control, Day 5 | 0.0338 |
| Virus, Day 1 | >0.05 |
| Virus, Day 5 | >0.05 |
|  |  |
|  |  |
| **SBV comparisons** | |
| Mixed model ANOVA, df = 4, 44; F=2.28525; p=0.02329 | |
| Tukey HSD posthoc test | p-value |
| **Treatments vs Field Honey bees** |  |
| Control, Day 1 | p>0.05 |
| Control, Day 5 | p>0.05 |
| Virus, Day 1 | p>0.05 |
| Virus, Day 5 | p>0.05 |
| **Treatments vs Apiary** |  |
| Control, Day 1 | p>0.05 |
| Control, Day 5 | p>0.05 |
| Virus, Day 1 | p>0.05 |
| Virus, Day 5 | p>0.05 |
